# Visual and automatic classification of the cyclic alternating pattern in electroencephalography during sleep

**DOI:** 10.1590/1414-431X20188059

**Published:** 2019-02-25

**Authors:** R. Largo, M.C. Lopes, K. Spruyt, C. Guilleminault, Y.P. Wang, A.C. Rosa

**Affiliations:** 1 LaSEEB - Evolutionary Systems and Biomedical Engineering Laboratory, Institute for Systems and Robotics (ISR-Lisboa), Instituto Superior Técnico (IST), University of Lisbon, Lisbon, Portugal; 2 Instituto de Psiquiatria (PRATA), Hospital das Cl�nicas (HCFMUSP), Faculdade de Medicina, Universidade de São Paulo, São Paulo, SP, Brasil; 3 Lyon Neuroscience Research Center, INSERM U1028-CNRS UMR 5292 Waking Team, School of Medicine, University Claude Bernard, Lyon, France; 4 Sleep Disorders Clinic, Stanford University Medical Center, Stanford, CA, USA; 5 Instituto de Psiquiatria (LIM-23), Hospital das Clinicas (HCFMUSP), Faculdade de Medicina, Universidade de São Paulo, São Paulo, SP, Brasil; 6 Escola Superior de Tecnologia de Setúbal, Instituto Politécnico de Setúbal, Setúbal, Portugal

**Keywords:** Cyclic alternating pattern, Sleep, Visual scoring, Automatic scoring

## Abstract

Cyclic alternating pattern (CAP) is a neurophysiological pattern that can be visually scored by international criteria. The aim of this study was to verify the feasibility of visual CAP scoring using only one channel of sleep electroencephalogram (EEG) to evaluate the inter-scorer agreement in a variety of recordings, and to compare agreement between visual scoring and automatic scoring systems. Sixteen hours of single-channel European data format recordings from four different sleep laboratories with either C4-A1 or C3-A2 channels and with different sampling frequencies were used in this study. Seven independent scorers applied visual scoring according to international criteria. Two automatic blind scorings were also evaluated. Event-based inter-scorer agreement analysis was performed. The pairwise inter-scorer agreement (PWISA) was between 55.5 and 84.3%. The average PWISA was above 60% for all scorers and the global average was 69.9%. Automatic scoring systems showed similar results to those of visual scoring. The study showed that CAP could be scored using only one EEG channel. Therefore, CAP scoring might also be integrated in sleep scoring features and automatic scoring systems having similar performances to visual sleep scoring systems.

## Introduction

Cyclic alternating pattern (CAP) in non-rapid eye movement (NREM) sleep is a neurophysiological pattern that can be visually scored by international criteria, and has been described in several conditions over a period of 20 years (1). Visual CAP scoring is a new way to measure sleep instability, and it could be a feasible tool to detect changes in brain plasticity. Some authors have been describing the essential roles of spontaneous brain activity in basic brain functioning and in the understanding of the working mechanisms of the brain (2,3). In fact, the sleep slow waves are homeostatically regulated and linked to learning and plasticity processes (4). The CAP pattern may therefore be a feasible marker of sleep instability showing the mechanisms involved, which allows a better understanding of neural plasticity during sleep (5). Moreover, this scoring system allows recognition of sleep arousals and disturbances that are not scored with the international criteria for scoring the sleep macrostructure.

CAP analysis has been performed by detection of electrocortical events with regular intervals in a range of seconds during NREM sleep (6). These events may be clearly distinguishable from the background electroencephalogram (EEG) rhythm as abrupt frequency shifts or amplitude changes. Two phases (A and B) are present as part of a CAP cycle and recur within 2 to 60 s (7). When neither of the phases (A and B) is identifiable, sleep has reached a new stable non-CAP (NCAP) state. The identification of CAP during sleep allows a different approach to investigate NREM sleep in many sleep disorders. It allows the recognition of disturbances of NREM sleep that are not visually identified using either the international manual of Rechtschaffen and Kales (8) or the American Sleep Disorders Association scoring criteria (9) for the identification of short (3 s or longer) EEG arousals (10). CAP has also been useful in the investigation of normal sleep (11,12), sleep changes by acoustic perturbations (13), cardiorespiratory perturbations (14–16), and neurological conditions (17,18). It advances our understanding of the physiologic components of sleep (19–23) and normal changes occurring during nocturnal sleep (24–26).

One limitation for the application of CAP analysis in sleep medicine has been the extra time needed to apply such a scoring system in clinical practice. Namely, the classification of short duration events in sleep EEG like the A phases of CAP is a laborious and error-prone task. Nonetheless, the clinical meaning has been linked to the detection of sleep instability in mild sleep disorders (5). The effects of age, patient group, and recording characteristics on the scoring agreement, however, are not completely known (27–31). For instance, studies investigating specific medical syndromes call upon age-matched controls run in the same laboratory. Therefore, there is great interest in developing an automatic scoring system that could be useful in clinical practice.

Another limitation is the concern about the feasibility, accuracy, and reliability of CAP classification independent of sleep stages using a single central EEG lead (C4-A1 or C3-A2). According to Rosa et al. (32), CAP scoring can be done with only one channel of EEG, and the evaluation of the inter-scorer agreement in a variety of recordings was useful in comparison with an automatic scoring system.

The main objective of this work was to assess the viability and performance of visual and automatic scoring on a single EEG channel, as suggested by the published CAP scoring rules in Terzano et al. (1), using recordings from different laboratories of subjects with different age, gender, health condition, and sampling frequency.

## Material and Methods

Sixteen hours of single-channel European data format (EDF) in eight polysomnographic recordings from four different sleep laboratories with either C4-A1 or C3-A2 channel and with different sampling frequencies (100, 128, and 256 Hz) were used. In order to speed up the visual classification process, 2 h of NREM sleep were extracted from each recording, 1 h epochs of NREM from the first half and the other 1 h epochs from the second half of the night, totaling 16 files.

### Methods

Seven scorers (G, N, P, Q, X, Z, and Y) performed visual scoring according to CAP scoring criteria described in Terzano et al. (1), and two automatic scoring systems (V and A) were used in the study. All scorers were blind to any information on each selected segment. Five visual and one automatic CAP scoring was performed using Somnologica Science 3 software version 3.3.2 (Flaga Hf, Iceland). The automatic scoring “A” is described in Rosa et al. (33) and “V” is fully described in Largo et al. (34). All the phase A scorings were converted into International Standards Organization (ISO) time format and converted to the same sampling frequency (when appropriate) for agreement analysis (35).

### Analysis

Two types of analysis were performed: the first was the time-based agreement of the scorings, where the scorings were compared for every sampling point and the second was event-based, where an agreement was scored whenever 2 events have an intersection longer than 0.5 s. In this report, only the event-based analysis is presented.

**Figure 1:**
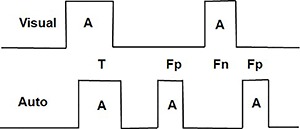
Event-based scoring agreement analysis. Whenever two events (A) intersect for more than a specific period, it is considered a hit (T). Other events are marked either as a miss (Fn: False negative) or a false (Fp: False positive).

Figure 1 shows the different possibilities for the event-based analysis: when 2 events intersect for more than a specific period of time, this is considered a “hit” (T: True), otherwise events are marked either as a “miss” (Fn: false negative) or as a “false” (Fp: false positive). As an example, in Figure 1, if we take the top trace (visual) as the reference, we have one T, one Fn, and 2 Fp.

Following the above example, a specific agreement analysis can be proposed as shown in Table 1. The “mutual agreement” (MA) is the measure of the agreement between any two scorers without taking either of them as the reference (36). We can only calculate the sensitivity of scorer 2 in relation to reference scorer 1 (SS) and the positive predictive value (PP). The number of disagreements (false negatives and false positives) per minute can be an alternative index.

**Table 1:** Truth table for events scoring.

	Phase A (1=Ref)	Phase B (1=Ref)	
Phase A (2)	T	Fp	Positive Predictive value=T/(T+Fp)
Phase B (2)	Fn	(Fp+Fn)/2	
	Sensitivity=T/(T+Fn)		Mutual Agreement MA=T/(T+(Fn+Fp)/2)

Ref: reference; 1: Scorer 1; 2: Scorer 2; T: true positive; Fp: false positive; Fn: false negative; MA: mutual agreement.

## Results

The total number of events scored by the 2 automatic scoring systems and the 7 visual scorers are presented in Table 2. Their values were between 939 and 2036. All pairwise scorers (automatic or visual) were compared file-by-file and the average results of MA are reported in Table 2. The table is symmetrical, therefore only the upper triangular matrix is presented. The inter-scorer MA values were between 55.5 % (AP) and 84.3% (VA). The average MA for each scorer compared with all the others is presented in Table 2, and shows a minimum of 60.6% and a maximum of 73.5%. The global average was 69.9% with standard deviation of 6.9%

**Table 2:** Inter-scorers mutual agreement.

	Scorer by scorer MA%								Average MA%	# Events
	a	g	n	p	q	X	z	Y		
v	84.3	71.1	73.9	59.7	73.1	80.8	78.4	66.3	73.5	1882
a	.	68.0	69.5	55.5	69.1	76.2	76.3	62.4	70.2	1864
g		.	75.5	60.6	71.6	70.9	74.1	63.4	69.4	1693
n			.	61.0	77.2	76.0	73.2	70.3	72.1	1689
p				.	65.3	58.0	58.5	66.2	60.6	939
q					.	74.9	74.4	73.4	72.4	1430
x						.	75.1	65.6	72.2	2036
z							.	67.8	72.2	1625
y								.	66.9	1126

Scorer by scorer mutual agreement (MA%) table is symmetrical; therefore, only the upper matrix without diagonal is presented. The last two columns are average MA% and total number of events for each scorer.

**Figure 2:**
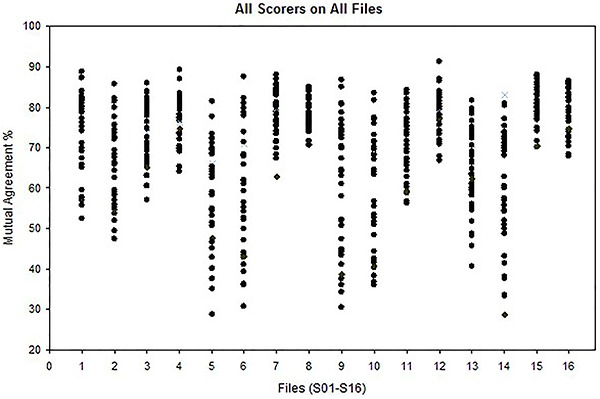
Scatter plot of all mutual agreement (%) values in different files.

**Figure 3:**
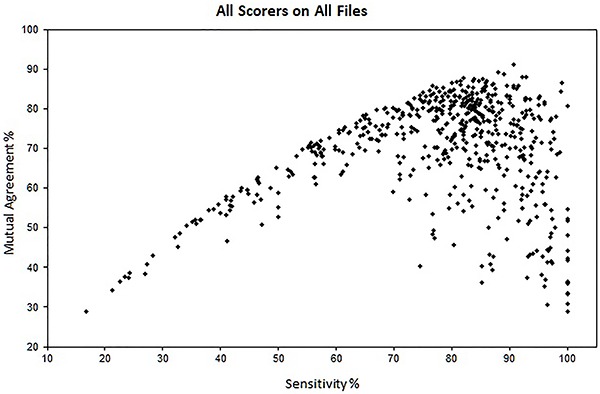
Mutual agreement scatter plot versus sensitivity for all pairwise file-by-file comparisons. This graph shows high density values in 80% neighborhood.

**Figure 4:**
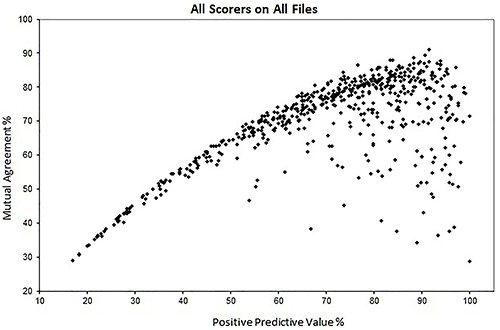
Mutual agreement scatter plot versus positive predictive value for all pairwise file-by-file comparisons.

**Figure 5:**
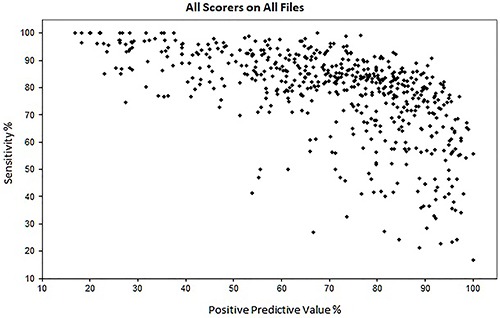
Sensitivity scatter plot versus positive predictive value for all pairwise file-by-file comparisons.

We calculated the MA between laboratories (Figure 2). The sensitivity for all pairwise file-by-file comparisons showed high density values around 80%. The limit curve of this graph suggested a “Pareto front” for the scoring process, indicating also that the best averaged MA sensitivity was 90%. The sensitivity was compared to PP value for all pairwise file-by-file comparisons (Figure 3). Figure 4 shows the MA scatter plot versus PP value for all pairwise file-by-file comparisons and is very similar to Figure 3. This was expected because the comparison was not made with a reference but between two scorers. Figure 5 shows a scatter plot of sensitivity versus PP value for all pairwise file-by-file comparisons. The PP value for all pairwise file-by-file comparisons was generated. This was expected because the comparison was not made with a reference but between two scorers. The inter-scorer MA for all files was above 80%; as an example, the comparison between two of the most experienced scorers was 87% MA.

All file averages are reported in Table 3. The upper triangle shows the sensitivity values that were between 43.3 and 91.4%. The lower triangle shows the positive predictive values that were between 42.6 and 94.2%. Average values for each scorer compared with all the others are reported in the last column and last line. The global average and the standard deviation were 78.1 and 11.5%, respectively, for sensitivity, and 70.0 and 12.7%, respectively, for PP values.

**Table 3:** Inter-scorers average sensitivity (SS%) and positive predictive values (PP%).

SS% PP%	v	a	g	N	p	q	x	z	y	Average SS%
v	.	84.9	80.8	81.2	90.7	85.6	80.9	85.9	87.7	84.7
a	83.9	.	78.2	77.5	88.4	82.2	76.4	83.9	85.6	82.1
g	68.2	65.0	.	77.6	83.9	78.4	67.1	75.7	82.4	78.0
n	70.6	65.8	79.8	.	88.9	84.2	71.4	75.9	87.4	80.5
p	47.0	42.6	54.4	49.1	.	54.4	43.3	48.0	58.8	69.6
q	66.4	62.2	72.2	73.5	86.4	.	66.2	72.4	83.8	75.9
x	82.1	77.5	82.9	84.1	94.2	88.8	.	84.7	91.4	72.7
z	73.7	71.2	78.1	75.4	84.5	80.7	69.4	.	86.1	76.6
y	56.5	52.1	61.1	62.5	77.1	67.3	53.5	60.1	.	82.9
Average PP%	68.6	65.0	70.2	70.1	66.9	74.7	79.1	74.1	61.3	

All files average sensitivity (upper triangle) and positive predictive values (lower triangle). Last column and last line represent inter-scorers average sensitivity and positive predictive values.

**Figure 6:**
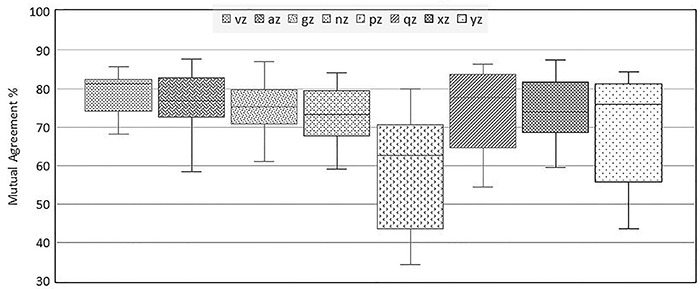
Box plot with all files inter-scorer mutual agreement with scorer Z. The boxes are between 1st and 3rd quartile. The interquartile range is between 20 and 50%. The line inside is the median. Scorers: vz, az, gz, nz, pz, qz, xz, and yz.

Figure 6 shows a box plot of the inter-scorer MA for all files with scorer Z considered as the most experienced laboratory for CAP scoring. The boxes are between 1st and 3rd quartile. The interquartile range is between 20 and 50%. The line inside is the median. It is interesting to note that for many scorers the box is mainly around the interval 60–80%, with the median between 70 and 80%. These examples may suggest that the identification of the point where a phase A ends was, at times, difficult. Likewise, for the issue of visual recognition of change, not only in amplitude but also in frequency, delineating CAP has been a source of difficulties when using a single-channel EEG. However, in spite of these difficulties, the results were often closely related.

## Discussion

**Figure 7:**
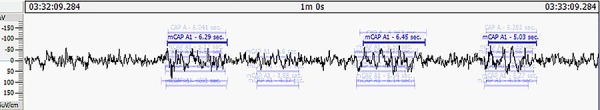
Sleep stage N3, one-minute epoch scorings. We found agreement between scorers.

**Figure 8:**
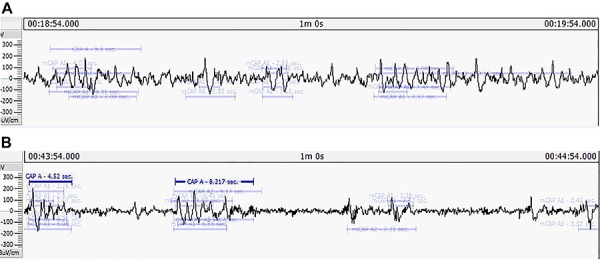
Sleep stage N2, one-min epoch scorings. We found disagreement between scorers (**A** and **B**).

This is the first study to show that CAP can be scored using only a single EEG channel. Although the CAP scoring experience of different scorers varied considerably (from newcomer to very experienced), the results were in ranges seen for inter-scorer agreement in similar studies (32). An interesting finding in our study was that the automatic scoring system (scorer V) had the highest overall MA (73.4%) and sensitivity (84.9%). The examples reported in Figures 7 and 8 show some of the difficulties encountered in visual scoring. There were inconsistencies in the CAP patterns selected by any given subject when such visual difficulty occurred, but the automatic system was consistent in its choices, based on amplitude and frequency criteria.

Our results demonstrated that the automatic analysis of CAP has a clinical application. Because the number of events to be visually scored was usually large (several hundred up to thousands), it was necessary to mark the events boundary accurately, otherwise the classification of the events into different A subtypes and the precision of the phase durations, cycles, and alternating conditions could be compromised. There is already some published data on visual CAP scoring agreement with automatic scoring systems (37,38) for events such as arousals (39) or CAP A phases. However, besides the overall values of correctness, specificity, and sensitivity, these studies only report detailed information on the detection method. The analysis of the performance of the automatic scoring system was done on a limited number of tracings and in a limited part of the night. The reference scoring, furthermore, has been described by one or two experts. The inter-scorer reliability in scoring CAP parameters of normal sleep has only been evaluated in two papers (32,40) and was based on only a few scorers, while intra-lab and automatic scoring is evaluated in Rosa et al. (32). In Ferri et al. (40), 4 human scorers participated in the study and reported values of the Kendall W coefficient of concordance above 0.9 for total CAP time, CAP time in sleep stage 2, and percentage of A phases in sequence; the CAP rate also showed a high value.

Moreover, ambulatory monitoring is increasingly performed in clinical practice and often one EEG lead is the only available EEG information. Our results showed that just one EEG lead was well correlated between human and automatic scoring. Development of automatic scoring will allow greater usage of CAP scoring in clinical practice, particularly when using ambulatory monitoring with few recording leads. Considering the potential large clinical usage of such automatic scoring systems and our encouraging results, there is no doubt that CAP scoring could be easily integrated into conventional sleep scoring. Our findings further suggest that usage of a single EEG channel for automatic scoring could be more refined by using visual analysis of several EEG leads.

Some limitations of the CAP analyses, however, have to be addressed, particularly the clinical meaning of the CAP rate. For now, it has only been proposed as a NREM sleep tool to evaluate sleep instability. Probably due to the amplitude criteria, it is not applied to REM sleep. However, the automatic scoring of CAP following the default decisions made by specialists in CAP analyses may assist the users in improving their recognition of NREM sleep. In conclusion, once several NREM sleep disturbances can be assessed by CAP analyses, the automatic scoring system or CAP might be applied for research purposes to better understand sleep phenomena.
